# Evacuation at Home Delayed the First Medical Intervention in Minamisanriku Town after the 2011 Great East Japan Earthquake

**DOI:** 10.1017/S1049023X2300050X

**Published:** 2023-06

**Authors:** Motohiro Tsuboi, Hiroyuki Sasaki, Hyejeong Park, Yuichiro Usuda, Makoto Hanashima, Masaji Saito, Shoko Takahashi, Kayako Sakisaka, Manabu Hibiya, Kazuya Kiyota, Kazuaki Hatsugai, Masafumi Nishizawa, Yumi Sugawara, Ichiro Tsuji, Shinichi Egawa

**Affiliations:** 1. International Cooperation for Disaster Medicine Lab., International Research Institute of Disaster Science (IRIDeS), Tohoku University, Miyagi, Japan; 2. Department of Emergency and Critical Care Medicine, Japanese Red Cross Saitama Hospital, Saitama, Japan; 3. Disaster Medical Informatics Lab., IRIDeS, Tohoku University, Miyagi, Japan; 4. National Research Institute for Earth Science and Disaster Resilience (NIED), Ibaraki, Japan; 5. Minamisanriku Hospital, Miyagi, Japan; 6. Minamisanriku Town, Miyagi, Japan; 7. Faculty of International Liberal Arts, Kaichi International University, Chiba, Japan; 8. Teikyo Academic Research Center, Teikyo University, Tokyo, Japan; 9. Department of Epidemiology, Department of Public Health and Forensic Medicine, Tohoku University Graduate School of Medicine, Miyagi, Japan

**Keywords:** disaster health management, disaster-related indirect death, evacuation at home, evacuation center, health resilience

## Abstract

**Introduction:**

In Japan, evacuation at home is expected to increase in the future as a post-disaster evacuation type due to the pandemic, aging, and diverse disabilities of the population. However, more disaster-related indirect deaths occurred in homes than in evacuation centers after the 2011 Great East Japan Earthquake (GEJE). The health risks faced by evacuees at home have not been adequately discussed.

**Study Objective:**

This study aimed to clarify the gap in disaster health management for evacuees at home compared to the evacuees at the evacuation centers in Minamisanriku Town, which lost all health care facilities after the 2011 GEJE.

**Methods:**

This was a retrospective cross-sectional and quasi-experimental study based on the anonymized disaster medical records (DMRs) of patients from March 11 through April 10, 2011, that compared the evacuation-at-home and evacuation-center groups focusing on the day of the first medical intervention after the onset. Multivariable Cox regression analysis and propensity score (PS)-matching analysis were performed to identify the risk factors and causal relationship between the evacuation type and the delay of medical intervention.

**Results:**

Of the 2,838 eligible patients, 460 and 2,378 were in the evacuation-at-home and evacuation-center groups, respectively. In the month after the onset, the evacuation-at-home group had significantly lower rates of respiratory and mental health diseases than the evacuation-center group. However, the mean time to the first medical intervention was significantly delayed in the evacuation-at-home group (19.3 [SD = 6.1] days) compared to that in the evacuation-center group (14.1 [SD = 6.3] days); P <.001). In the multivariable Cox regression analysis, the hazard ratio (HR) of delayed medical intervention for evacuation-at-home was 2.31 with a 95% confident interval of 2.07–2.59. The PS-matching analysis of the adjusted 459 patients in each group confirmed that evacuation at home was significantly associated with delays in the first medical intervention (P <.001).

**Conclusion:**

This study suggested, for the first time, the causal relationship between evacuation at home and delay in the first medical intervention by PS-matching analysis. Although evacuation at home had several advantages in reducing the frequencies of some diseases, the delay in medical intervention could exacerbate the symptoms and be a cause of indirect death. As more evacuees are likely to remain in their homes in the future, this study recommends earlier surveillance and health care provision to the home evacuees.

## Introduction

The Sendai Framework for Disaster Risk Reduction 2015-2030^1^ and World Health Organization (WHO; Geneva, Switzerland) Health Emergency and Disaster Risk Management Framework^2^ reported that quantitative risk management and the establishment of appropriate health support systems in the acute phase are important for strengthening disaster resilience to mitigate health effects during a large-scale disaster. The health effects of disasters can be broadly classified into direct and indirect effects, with disaster deaths being the most important endpoint to prevent.^3–6^ Although it is important to reduce direct deaths by natural hazards such as earthquakes, tsunamis, floods, and storms, it is critical to prevent indirect deaths caused by delays in medical intervention. In Japan, post-disaster indirect deaths have been noted since the 1995 Great Hanshin-Awaji Earthquake.^3^ In the 2011 Great East Japan Earthquake (GEJE) that occurred on March 11, there were 3,786 indirect deaths as of January 2023, and approximately 60% of these deaths occurred within one month after the disaster, indicating a large indirect impact in the acute phase.^3^ Furthermore, 14% of the deaths that occurred within one month after the disaster were preventable disaster deaths that took place mainly due to the poor evacuation environment and the lack of a health care support system in the acute phase.^6^ Therefore, since the GEJE, improvements have been made to the evacuation center environment and to the health care support system in Japan, focusing on evacuation centers.^3^ However, a survey by the Reconstruction Agency (Tokyo, Japan) reported that 46% of indirect deaths in the GEJE occurred at home, much higher than the proportion of those that occurred in evacuation centers (18%).^7^ There could be a lack of an organized system to protect the health of evacuees who are evacuated at home.

A PubMed (National Center for Biotechnology Information, National Institutes of Health; Bethesda, Maryland USA) search for “disaster,” “evacuation,” “at home” regarding home evacuation during a disaster identified 25 articles as of January 2023, out of which three were relevant articles, including this study.^8–10^ Due to the recent COVID-19 pandemic, aging of the population, and the diversity of disabilities, evacuations outside of evacuation centers, mainly evacuation at home, will increase in the future for large-scale disasters in Japan.^8^ A previous study reported increased frequencies of common cold and diarrhea in the patients treated in the evacuation centers in Minamisanriku Town than the usual prevalence.^9^ Moreover, staying in evacuation centers is a risk factor for sleep disturbance.^10^ However, no previous studies have addressed the challenges related to health care support systems for evacuation at home.

In addition, the systematic review list of EvidenceAid (Weymouth, Dorset, UK) was searched with the keyword “evacuation” to find systematic reviews on evacuation behavior and health aspects of evacuees.^11–20^ Among them, Ochi, et al reported that the loss of medicines can exacerbate the health outcomes of evacuees.^15^ Various type of non-communicable diseases (NCDs) and mental health issues after the cyclones and the 2011 GEJE were highly reported,^17,18^ suggesting the change of health needs in the aging society, but no systemic review article has addressed health care systems for evacuation at home. Recently, extreme weather conditions led to affected people being evacuated at home or made it difficult for patients at home to evacuate to the designated evacuation shelters.^21^ There could have been isolated high-risk individuals who were unable to, or did not go to evacuation centers. Therefore, this study hypothesized that evacuation at home has health risks for better health care management in disasters.

**Figure 1.**
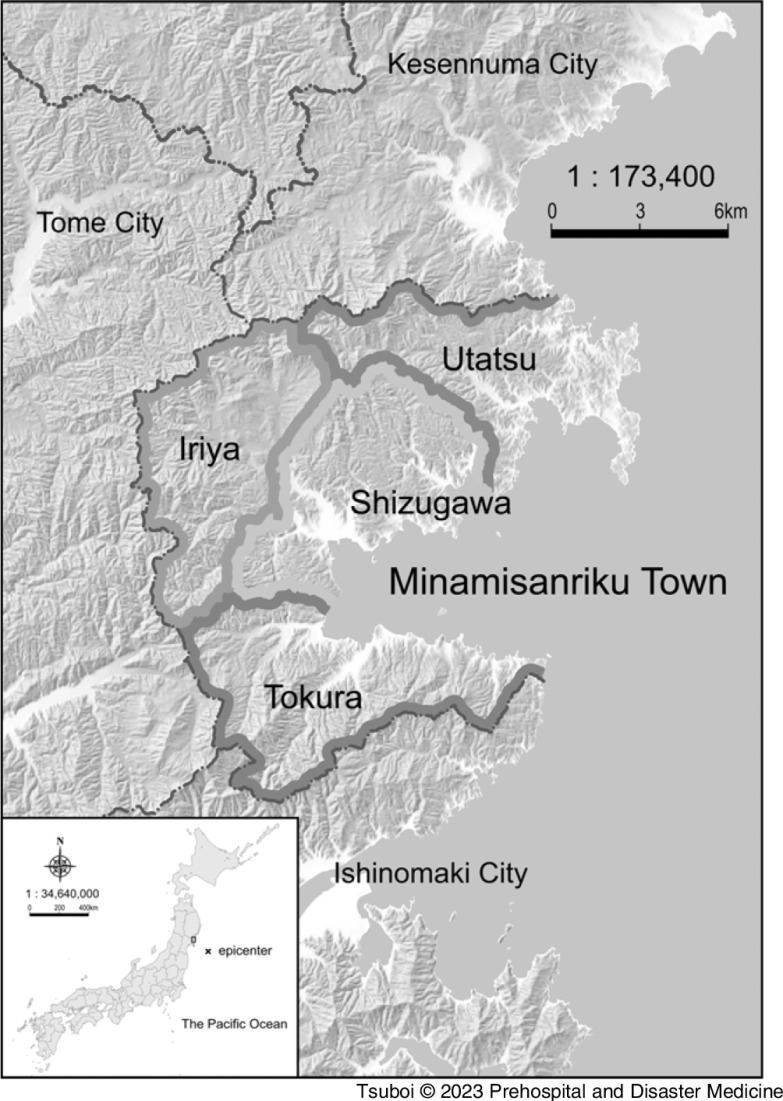
Location of Minamisanriku Town, Miyagi Prefecture, Japan. Note: The Minamisanriku Town is subdivided into four districts, Shizugawa (downtown), Utatsu, Iriya, and Tokura.

This study aimed to examine whether evacuation at home delayed the first medical intervention in Minamisanriku Town, Miyagi Prefecture, which was severely damaged by the GEJE and lost all existing medical facilities in the area due to the tsunami^9,10^ (Figure 1).

## Methods

A retrospective cross-sectional study was conducted using the database of anonymized disaster medical records (DMRs) of Minamisanriku from March 11, 2011 through April 10, 2011, after the GEJE.^9^ Cox-regression analysis was used to estimate the association between age, sex, disease classification, disease modules (wider classification^9^), and subdistrict in relation to the first medical intervention. This study conducted a propensity score (PS)-matching analysis as a quasi-experimental study^22^ to adjust the baseline characteristics between the two evacuation types to estimate the causal effect of evacuation types on the time until first medical intervention.

### Establishment of the Database

As previously described,^9^ a company that acquired PrivacyMark (JIPDEC JIS Q 15001:2006, ID 23820061; Tokyo, Japan) concealed the confidential personal information from handwritten DMRs and converted each anonymized DMR into a PDF file. The PDF file was imported as an image into a database (FileMaker Pro; Claris, Inc., Tokyo, Japan). Patient data, including age, sex, evacuation type, date of consultation, diagnosis, date of diagnosis, symptom, treatment, and prescription, were input manually. The location of the treatment (regardless of evacuation at home or evacuation center) was automatically assigned a subdistrict according to the place mentioned in the DMR. The diagnoses were automatically classified into five modules: NCD, infectious disease, mental health issues, trauma, and mother and child health issues.^9^

This study considered one anonymized DMR (one PDF) as a record of one patient after removing apparent duplicates, although it was impossible to eliminate duplicates completely because of anonymity. The day of first medical intervention was defined as the first recorded day of consultation for any medical assistance in the evacuation center, home, or field clinics, although some records lack the information of the date of possible earlier consultation. This study counted the first appearance of a diagnosis in the DMR as the onset of the disease, irrespective of prior history.

### Inclusion Criteria

This study included the following anonymized DMRs for the analysis:^9^

1. DMRs on the date of the first consultation from March 11, 2011 (Day 0) through April 10 (Day 30), 2011;
2. DMRs with at least one date of consultation (a day when the patient met a medical team), the earliest recorded date of consultation was adopted as the first medical intervention; and
3. DMRs identifiable as “evacuation at home” or “evacuation center” as the evacuation type.

### Exclusion Criteria

The following documents were excluded:

1. DMRs with no date of consultation;
2. Duplicate copies of a record;
3. Record of death diagnosis;
4. Documents other than DMRs;
5. Documents with information about multiple patients;
6. Patients with no information of evacuation types; and
7. Patients with no information on age or sex or subdistrict.

### Cross-Sectional Analysis

A cross-sectional study was conducted including all eligible patients in the analysis. This study conducted a univariable analysis of the patient characteristics of the evacuation-at-home and evacuation-center groups. A multivariable Cox-regression analysis (forced entry method) was used to estimate the hazard ratio (HR) for the consultation rate of evacuation at home within 30 days of the disaster, with evacuation centers as the reference. The covariates were age, sex, module, disease classification, and subdistrict. Accordingly, a forest plot compared the effect of evacuation type for the delay of the first medical intervention in each subgroup by age, sex, and module.

### PS-Matching Analysis

A logistic regression model was applied to estimate the PS for each patient, adjusting the background characteristics of age, sex, module, disease classification, and subdistrict in the evacuation-at-home and evacuation-center groups. Balance between the evacuation types was assessed with the standardized mean differences (SMD). An SMD of 0.1 or less was deemed to be the ideal balance, and an SMD of 0.2 or less was deemed to be an acceptable balance.^23^ In the PS-matched population, Kaplan–Meier curves were generated for the time to first medical intervention in each group, and a log-rank test was used to test the statistical difference.

### Statistical Analysis

Categorical data were expressed as n (%), and continuous data were expressed as mean (standard deviation [SD]). The categorical data were compared using the χ-squared test, and continuous data were tested using the unpaired t-test. If the two-sided P value was less than .05, the analysis was considered statistically significant. Sample size calculation was not enforced. The data were analyzed using JMP 16 (SAS Institute Inc.; Cary, North Carolina USA).

### Ethical Considerations

This study was approved by the ethics committee of the Tohoku University Graduate School of Medicine (2021-1-1039; Sendai, Japan). Informed consent was not obtained from any patient because the DMRs were created during the disaster. Therefore, all DMRs were anonymized and digitized by the PrivacyMark company. As an anonymized database was used, this study protected the personal information of patients in accordance with “Ethical Guidelines for Medical and Health Research Involving Human Subjects.”^24^

## Results

### Patient Selection

**Figure 2.**
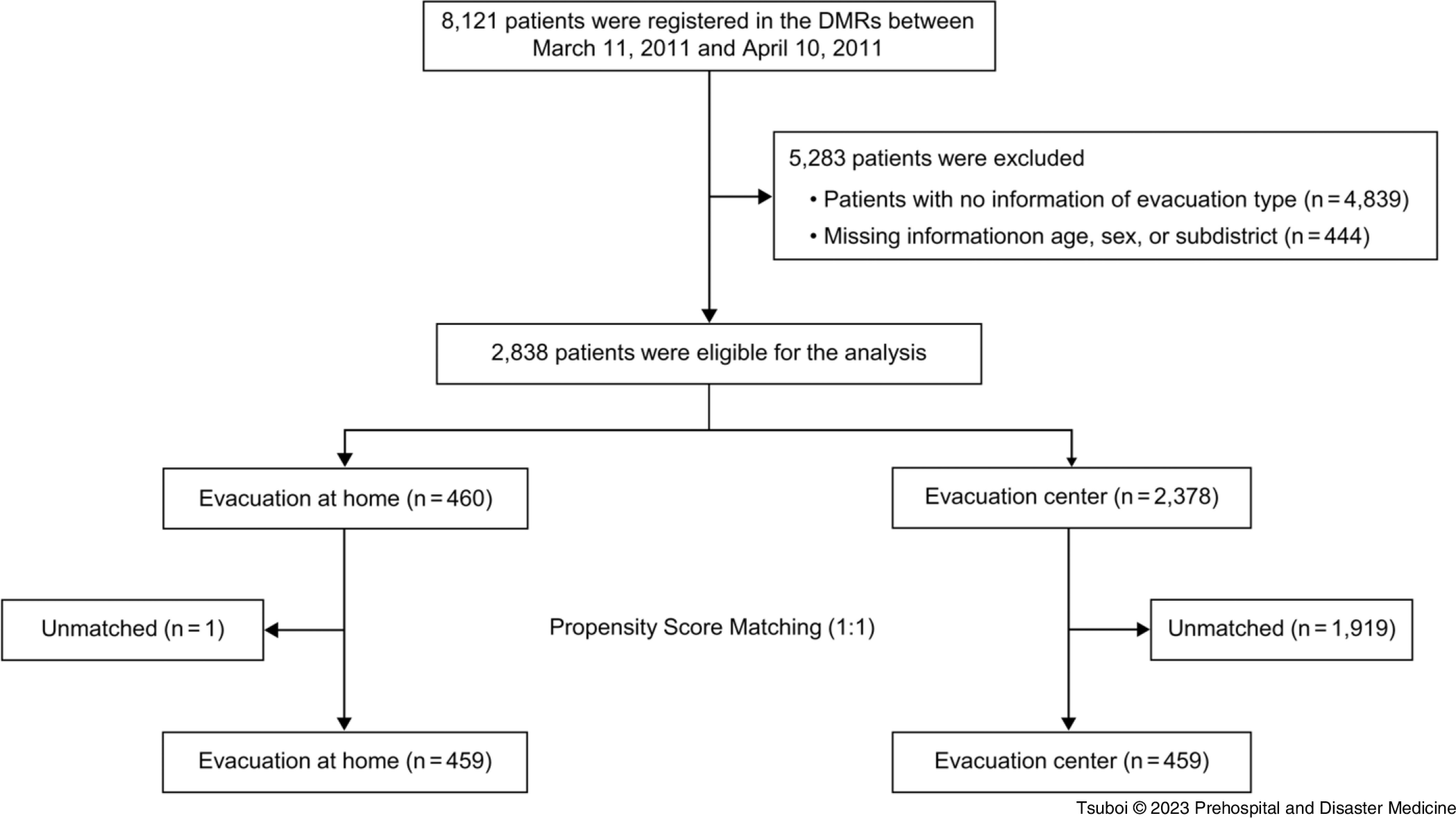
Flow Diagram of the Process of Patient Selection. Abbreviation: DMR, disaster medical record.

There were 8,121 patients in the anonymized DMR database from March 11 through April 10, 2011. According to the criteria, 4,839 patients with no information of evacuation type were excluded. Of the remaining 3,282 patients, 444 patients were further excluded because of missing information on age, sex, or subdistrict (Figure 2). Finally, 2,838 patients were included in the analysis and classified into the evacuation-at-home (n = 460) and evacuation-center groups (n = 2,378). The baseline characteristics of the overall study patients are summarized in Table 1.

**Table 1.** Baseline Characteristics of Patients in the Two Evacuation Types

Variables	Evacuation at Home (n = 460)	Evacuation Center (n = 2,378)	*P* Value
Patient Characteristics			
Age (mean [SD])	51.9 (SD = 24.6)	51.2 (SD = 24.0)	.112
Female Sex	260 (56.5%)	1,246 (52.4%)	.105
Module^a^			
NCD	378 (82.2%)	1,723 (72.5%)	<.001
Infectious Disease	164 (35.7%)	1,040 (43.7%)	<.001
Mental Health	36 (7.8%)	295 (12.4%)	.005
Trauma	28 (6.1%)	170 (7.1%)	.413
Disease Classification			
Respiratory Infection	126 (27.4%)	831 (34.9%)	<.001
Chronic Respiratory Diseases and Symptoms	27 (5.9%)	118 (5.0%)	.419
Cardiovascular Diseases and Symptoms	39 (8.5%)	132 (5.6%)	.016
Hypertension	123 (26.7%)	574 (24.1%)	.236
Stroke and Sequelae	7 (1.5%)	13 (0.5%)	.022
Cranial Nerve Diseases and Symptoms	4 (0.9%)	34 (1.4%)	.339
Metabolic Endocrine Disorders	114 (24.8%)	328 (13.8%)	<.001
Renal Disease	0 (0.0%)	2 (0.1%)	.534
Gastrointestinal Diseases and Symptoms	47 (10.2%)	215 (9.0%)	.425
Gastroenteritis/Diarrhea	49 (10.7%)	279 (11.7%)	.507
Mental Diseases Other than Sleep Disturbance	15 (3.3%)	148 (6.2%)	.012
Sleep Disturbance	24 (5.2%)	145 (6.1%)	.465
Dementia/Delirium	2 (0.4%)	16 (0.7%)	.556
Malignant Diseases	7 (1.5%)	7 (0.3%)	<.001
Ophthalmologic Diseases	26 (5.7%)	117 (4.9%)	.511
Oral and Maxillofacial Surgery Diseases	2 (0.4%)	28 (1.2%)	.154
Otolaryngology Diseases	0 (0.0%)	4 (0.1%)	.379
Pollinosis	97 (21.1%)	405 (17.0%)	.044
Male Urogenital Diseases	8 (1.7%)	26 (1.1%)	.244
Female Urogenital Diseases	6 (1.3%)	30 (1.3%)	.940
Pregnancy/Delivery/Postpartum	0 (0.0%)	2 (0.1%)	.534
Trauma	26 (5.7%)	156 (6.6%)	.467
Musculoskeletal Diseases and Symptoms	59 (12.8%)	265 (11.1%)	.300
Burns/Frostbite	2 (0.4%)	15 (0.6%)	.618
Skin Diseases and Symptoms	36 (7.8%)	158 (6.6%)	.358
Bedsore	2 (0.4%)	7 (0.3%)	.624
Subdistrict			
Shizugawa	446 (97.0%)	1,183 (49.7%)	<.001
Utatsu	8 (1.7%)	612 (25.7%)	
Tokura	3 (0.7%)	12 (0.5%)	
Iriya	3 (0.7%)	446 (18.8%)	
Out of Minamisanriku Town Boundary	0 (0.0%)	125 (5.0%)	
Time to First Medical Intervention (days)	19.3 (SD = 6.1)	14.1 (SD = 6.3)	<.001

Note: A patient may have multiple disease diagnoses, and accordingly, multiple modules. Therefore, the sum of the number of patients in disease classifications and modules may be different from the total number of patients in each group.

Abbreviation: NCD, non-communicable disease.

a Wider classification of diseases defined in Ref.^12^

### Cross-Sectional Analysis

Table 1 presents the univariable analysis of eligible patients between the evacuation-at-home (n = 460) and evacuation-center groups (n = 2,378) in Minamisanriku Town. The mean age was similar in the evacuation-at-home and evacuation-center groups at 51.9 (SD = 24.6) years and 51.2 (SD = 24.0) years, respectively. The proportion of women was slightly larger in the evacuation-at-home group than that in the evacuation-center group (56.5% versus 52.4%), but the difference was not statistically significant. Pre-disaster demographics in Minamisanriku Town indicated that the female population was slightly larger than the male population.^9^

The most frequent disease module was NCD, in accordance with the previous report,^9^ despite restricting the time of consultation to within one month after the onset and limiting the patients to the evacuation-at-home and evacuation-center groups. The NCD module was significantly more frequent in the evacuation-at-home group (82.2%) compared to that in the evacuation-center group (72.5%). Although the proportion of hypertension was similar in both the groups (26.7% versus 24.1%; P = .236), the metabolic endocrine disorders (24.8%), such as diabetes and hyperlipidemia, were more frequent in the evacuation-at-home group than in the evacuation-center group, followed by pollinosis (21.1%), cardiovascular diseases (8.5%), stroke and sequalae (1.5%), and malignant diseases (1.5%; Table 1).

In contrast, the infectious diseases module was significantly less frequent in the evacuation-at-home group compared to that in the evacuation-center group (35.7% versus 43.7%; P <.001). In particular, respiratory infections were significantly less frequent in the evacuation-at-home group (27.4% versus 34.9%; P <.001). The frequency of gastroenteritis/diarrhea was similar between the groups. The frequency of the mental health issues module was statistically less in the evacuation-at-home group compared to that in the evacuation-center group (7.8% versus 12.4%; P = .005). Mental diseases other than sleep disturbance were more frequent in the evacuation-center group (6.2% versus 3.3%; P = .012), whereas the frequency of sleep disturbance was similar. The first medical intervention was significantly delayed in the evacuation-at-home group at 19.3 (SD = 6.1) days compared to that in the evacuation-center group at 14.1 (SD = 6.3) days (P <.001).

**Figure 3.**
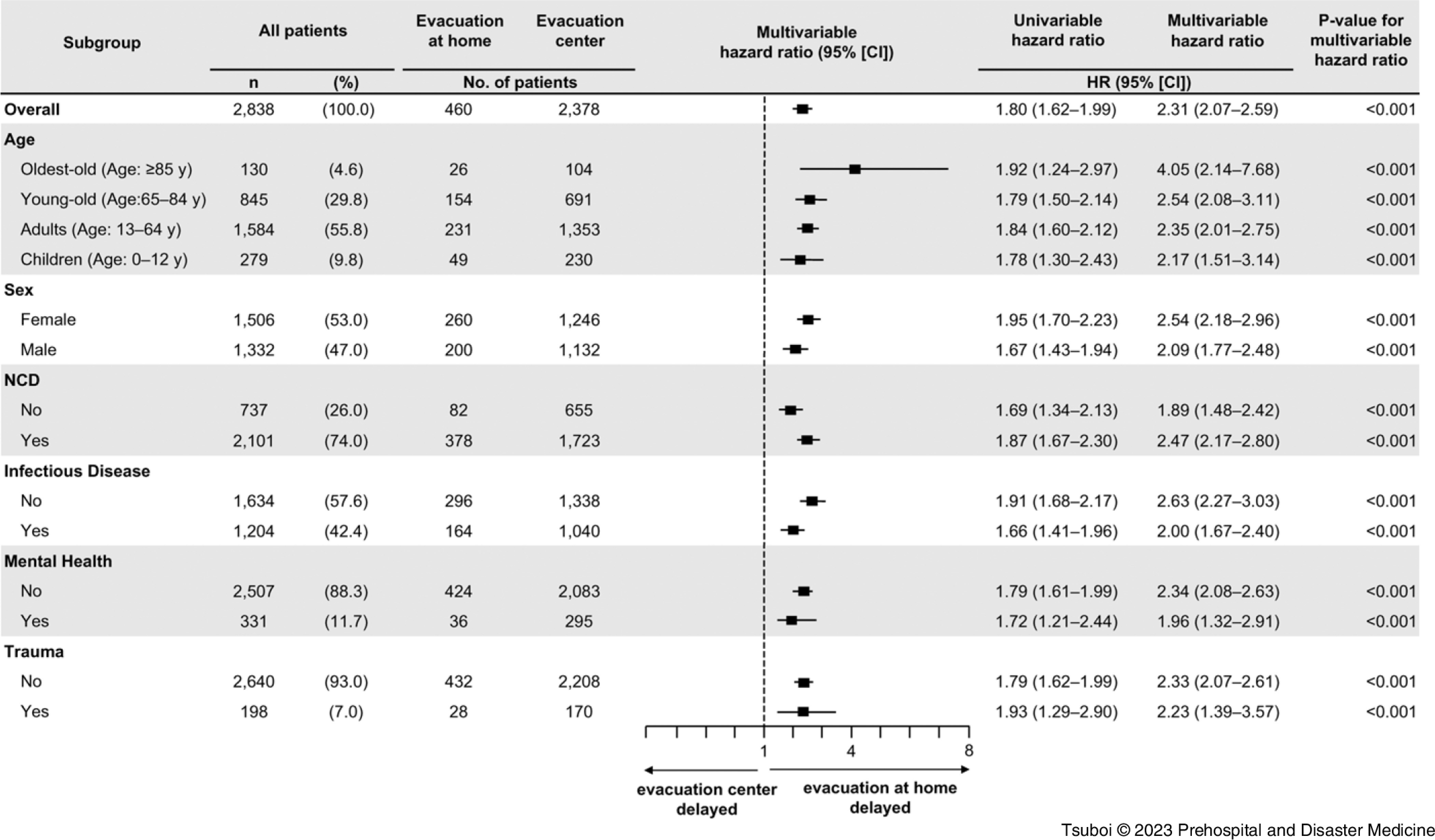
Forest Plot Analysis of Subgroups in Each Evacuation Type. Abbreviations: HR, hazard ratio; NCD, non-communicable disease.

Figure 3 shows the forest plot of the multivariable-adjusted Cox-regression analysis of time until the first medical intervention for the subgroups in the evacuation-at-home group compared to that of the evacuation-center group. Overall, the first medical intervention was delayed in the evacuation-at-home group compared to that in the evacuation-center group (HR = 2.31; 95% CI, 2.07–2.59). In addition, evacuation at home was associated with a delayed first medical intervention in all age, sex, and module subgroups.

### PS-Matching Analysis

**Figure 4.**
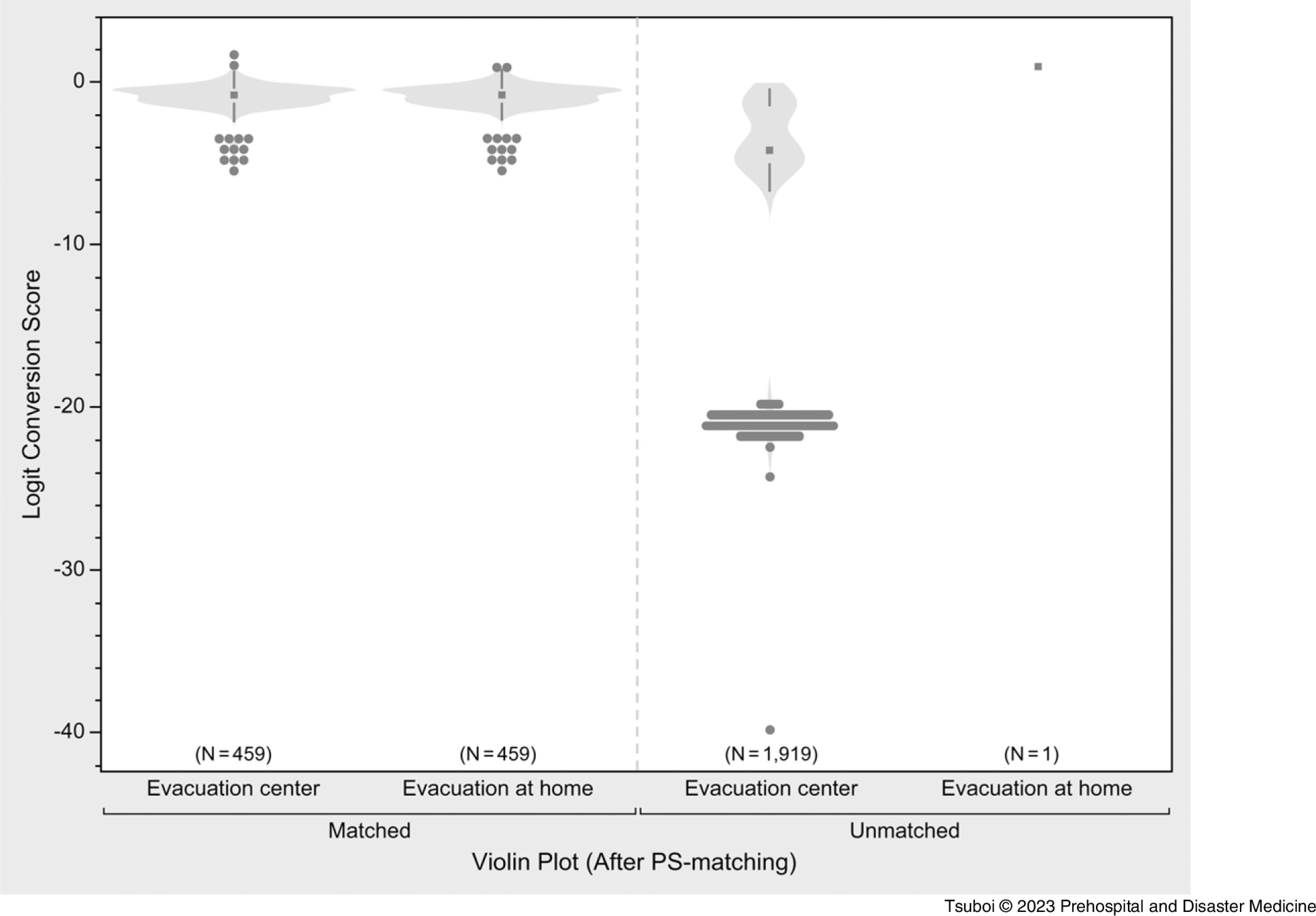
Violin Plot of Matched and Unmatched Populations by Propensity Score (PS) Matching.

**Figure 5.**
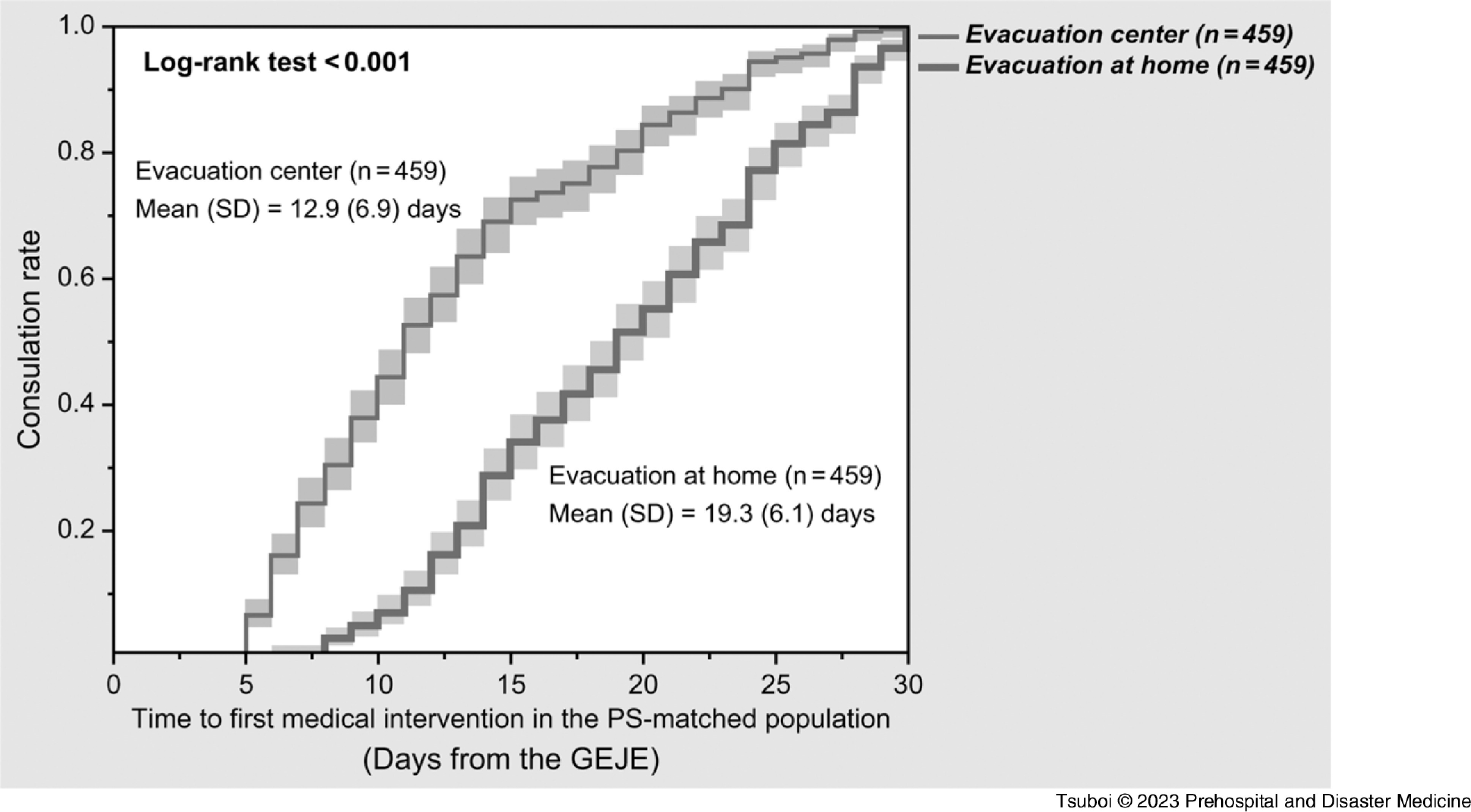
Kaplan–Meier Curves of Time to Medical Intervention for Evacuation Type Groups in PS-Matched Populations. Note: Bottom line = evacuation at home; top line = evacuation center. Pale squares indicate the 95% confidential intervals. Abbreviations: GEJE, Great East Japan Earthquake; PS, propensity score.

The variables used for PS estimation had a high accuracy for predicting time to medical intervention, with a C-statistics of 0.81.^25^ Through the matching process, 459 PS-matched pairs were generated (Figure 4). All the SMD values of the adjusted variables were <0.1, indicating a well-matched balance (Table 2).^23^ Using these matched groups, there was a significant delay until the first medical intervention in the evacuation-at home group compared to that in the evacuation-center group (19.3 days versus 12.9 days; P <.001); Figure 5.

**Table 2.** Propensity Score Matching by Age, Sex, Disease Module, and Disease Classification

Variables	Evacuation at Home (n = 459)	Evacuation Center (n = 459)	SMD	*P* Value
Patient Characteristics				
Age (mean [SD])	52.9 (SD = 24.6)	53.6 (SD = 23.7)	0.029	.781
Female Sex	259 (56.4%)	261 (56.9%)	0.010	.894
Module				
NCD	377 (82.1%)	368 (80.2%)	0.049	.448
Infectious Disease	164 (35.7%)	170 (37.0%)	0.027	.681
Mental Health	36 (7.8%)	32 (7.0%)	0.031	.614
Trauma	28 (6.1%)	28 (6.1%)	0.000	1.000
Disease Classification				
Respiratory Infection	126 (27.5%)	137 (29.8%)	0.051	.422
Chronic Respiratory Diseases and Symptoms	27 (5.9%)	26 (5.7%)	0.040	.888
Cardiovascular Diseases and Symptoms	39 (8.5%)	44 (9.6%)	0.038	.565
Hypertension	123 (26.8%)	136 (29.6%)	0.062	.340
Stroke and Sequelae	7 (1.5%)	7 (1.5%)	0.000	1.000
Cranial Nerve Diseases and Symptoms	4 (0.9%)	5 (1.1%)	0.020	.738
Metabolic Endocrine Disorders	114 (24.8%)	123 (26.8%)	0.046	.497
Renal Disease	0 (0.0%)	0 (0.0%)	0.000	–
Gastrointestinal Diseases and Symptoms	47 (10.2%)	45 (9.8%)	0.013	.826
Gastroenteritis/Diarrhea	49 (10.7%)	51 (11.1%)	0.013	.832
Mental Diseases Other than Sleep Disturbance	15 (3.3%)	13 (2.8%)	0.029	.701
Sleep Disturbance	24 (5.2%)	20 (4.4%)	0.037	.537
Dementia/Delirium	2 (0.4%)	3 (0.7%)	0.041	.654
Malignant Diseases	6 (1.3%)	3 (0.7%)	0.060	.315
Ophthalmologic Diseases	26 (5.7%)	20 (4.4%)	0.036	.364
Oral and Maxillofacial Surgery Diseases	2 (0.4%)	2 (0.4%)	0.000	1.000
Otolaryngology Diseases	0 (0.0%)	0 (0.0%)	0.000	–
Pollinosis	97 (21.1%)	92 (20.0%)	0.027	.683
Male Urogenital Diseases	8 (1.7%)	9 (2.0%)	0.022	.807
Female Urogenital Diseases	6 (1.3%)	8 (1.8%)	0.040	.590
Pregnancy/Delivery/Postpartum	0 (0.0%)	0 (0.0%)	0.000	–
Trauma	26 (5.7%)	26 (5.7%)	0.000	1.000
Musculoskeletal Diseases and Symptoms	59 (12.9%)	58 (12.6%)	0.09	.921
Burns/Frostbite	2 (0.4%)	2 (0.4%)	0.028	1.000
Skin Diseases and Symptoms	36 (7.8%)	37 (8.1%)	0.011	.903
Bedsore	2 (0.4%)	2 (0.4%)	0.000	1.000
Subdistrict				
Shizugawa	445 (97.0%)	445 (97.0%)	0.000	.968
Utatsu	8 (1.7%)	9 (2.0%)	0.009	
Tokura	3 (0.7%)	3 (0.7%)	0.000	
Iriya	3 (0.7%)	2 (0.4%)	0.041	
Out of Minamisanriku Town Boundary	0 (0%)	0 (0.0%)	0.000	
Time to First Medical Intervention (days)	19.3 (SD = 6.1)	12.9 (SD = 6.4)	1.024	<.001

Note: Standardized mean difference (SMD) to validate the adjustment. An SMD of 0.1 or less was deemed to be the ideal balance, and an SMD of 0.2 or less was deemed to be an acceptable balance.

Abbreviation: NCD, non-communicable disease.

## Discussions

This study showed, for the first time quantitatively, that evacuation at home delayed the first medical intervention compared to evacuation at evacuation centers in Minamisanriku Town after the GEJE. As per the univariable analysis (Table 1), the first medical interventions were significantly delayed in the evacuation-at-home group; however, the population of the evacuation-at-home group (n = 460) was far smaller than that of the evacuation-center group (n = 2,378), and there were many differences in the backgrounds of these two groups. The exploratory subgroup analysis also showed the delays in the first medical intervention in any subgroups of age, sex, and disease modules (Figure 3). The quasi-experimental PS-matching analysis showed the association between evacuation at home and delay in the first medical intervention, even after adjusting the background factors in both the groups (19.3 versus 12.9 days; P <.001); Figure 5. Thus, this study indicated the causal relationship between evacuation at home and delay in the first medical intervention. These results suggest the necessity to establish a health support system for evacuees at home in the acute phase who might receive less attention, despite the fact that a high number of indirect deaths occurred at home following the GEJE.^7^

This study shows the advantages of evacuation at home over that in evacuation centers, with the former being associated with a lower frequency of common cold (Table 1), probably due to the absence of the crowded environment of the evacuation center. Sleep disturbance, however, was not significantly different in each evacuation type; this result was inconsistent with that of the previous study.^10^ This study limited the diagnosis to a one-month duration after the onset and excluded many patients owing to the rigorous multivariable analysis and quasi-experimental model, and it could not show the benefit of evacuation at home on sleep disturbance. In contrast, mental disorders other than sleep disturbance, such as schizophrenia, anxiety disorder, and depression,^9,10^ were significantly more frequent in the evacuation-center group. There are two possible reasons: people with a prior history of mental disorder are preferentially evacuated to the evacuation center and there are patients with new onset and exacerbation of mental disorders, including posttraumatic stress disorder (PTSD), depression, and anxiety, in the evacuation center.^17^ Although the frequency in one month was less in the evacuation-at-home group, it does not assure the prevention of middle- to long-term occurrence of mental disorders.

The frequency of hypertension did not differ between the groups. Hypertension was the most frequent NCD in the DMRs of Minamisanriku Town after the GEJE.^9^ A systematic review reported that a considerable number of patients lose their medicines during evacuation, and many do not bring prescriptions with them while being evacuated.^15^ This study showed that the frequencies of cardiovascular diseases, stroke and sequelae, metabolic endocrine diseases, and malignant diseases were higher in the evacuation-at-home group (Table 1), suggesting that people with such conditions tend to stay at home, if possible. As reported, evacuees in evacuation centers were forced to live in poor shelter conditions, sleep directly on dusty floors, and live in crowded condition after the GEJE.^26,27^ Because similar pictures were broadcasted again and again since the 1995 Great Hanshin Awaji Earthquake, people of older age and those with chronic diseases and disabilities preferred to stay at home and needed health care support.

This study statistically revealed that, while there are definite needs and advantages of evacuation at home, the first medical intervention was delayed by one week. There are two aspects concerning the delay. The lack of understanding and surveillance of the medical needs of home evacuees can delay the first medical intervention for providing relief. As for the patient’s side, isolation, lack of information, and difficulties in mobility due to physical and mental conditions can delay the first consultation. Some people might refrain from consulting doctors until they notice the shortage of medicines. After the GEJE, all medical facilities in Minamisanriku Town were damaged due to the tsunami. Public health nurses, community welfare commissioners, administrative wardens, and home health care providers, who had been in close contact with the evacuees during normal times, tried to support the health of home evacuees through outreach by visiting door to door. They frequently noticed the reality of home evacuees living in a harsh environment, physical and psychological isolation due to the disruption of lifelines, delays in support, and lack of medical information compared to those in evacuation centers.^28^

Japan is currently facing the Nankai megathrust earthquakes and earthquake directly beneath the capital.^29^ In the event of a large-scale disaster in Japan in the future, evacuation at home will increase, and it is critical to understand and survey the health risks of people at home in the affected areas. In fact, in densely populated areas such as Tokyo, the number of evacuees will far exceed the capacity of shelters, and the government’s crisis management department recommends evacuation at home whenever possible.^30^ In a large-scale disaster, external emergency medical teams will majorly coordinate disaster health management together with the affected local stakeholders until the local health care providers recover.^31^ Ishii described the coordinated strategy for effective surveillance and assessment of evacuation centers in Ishinomaki City after the GEJE,^32^ which became a model of disaster health management in Japan. This study additionally reported the delay of the first medical intervention in the evacuation-at-home group, suggesting the importance of surveillance of evacuees at home as early as possible. The mean day of first medical intervention in the evacuation-center group was Day 14 before PS matching and Day 12 in the PS-matching analysis, suggesting that these numbers of days can be benchmarks of disaster health management.^33^ The improvement of inter-sectoral information exchange and preparedness to protect the health and welfare of affected people staying at home should be included in local disaster risk reduction plans.

## Limitations

There are several limitations of this study. First, the extent up to which the delay in the first medical intervention affected the health outcome of the home evacuees is unclear. Further studies are needed to determine the impact of delayed medical intervention on health and its relation to the indirect deaths. Second, the definition of evacuation type was based on the descriptions in the DMRs, and it was not possible to determine for how long people stayed at home or in evacuation centers. Furthermore, more than 4,000 records were excluded because of the lack of information on evacuation type, sex, and age. Thus, the effect of evacuation type could have been under-estimated or over-estimated. However, the large number of DMRs in this study made the multivariate analysis and PS matching possible and reduced the sampling bias on the finding.^22,23^ Third, there may be possible confounding factors that were not measured, such as oral medication use and individual medical history. Fourth, in Minamisanriku Town, support for planned evacuation at home was provided by local public health nurses, administrative wardens, community welfare committee members, and medical personnel, but the uneven distribution of support staff may have led to different results in areas where support and acceptance were delayed.

Although this study was not an interventional study, this quasi-experimental study using PS matching strongly suggested a causal relationship between post-disaster evacuation type and the first medical intervention. However, other regions must be analyzed as well due to the local context of disasters in order to generalize the results. In the future, analysis of data from other affected municipalities may provide more evidence to help reduce the reduction in medical accessibility to evacuation at home after a major natural disaster.

## Conclusion

This study quantitatively reported the risk of evacuation at home in a major disaster by a natural hazard for the first time. This study showed that evacuation at home was associated with delayed first medical intervention for all age groups, sexes, and module subgroups. In addition, the quasi-experimental study with PS matching provided strong evidence of a causal relationship between evacuation at home and delayed first medical intervention that might lead to worsening of the existing and the new onset of diseases. A health care support system must be established to improve the medical accessibility for evacuation at home, which will increase in the future.
